# The complete mitochondrial genome of heartbreak grass *Gelsemium elegans* (Gardner & Champ.) Benth. (Gelsemiaceae)

**DOI:** 10.1080/23802359.2019.1677525

**Published:** 2019-10-23

**Authors:** Yong-Hui Jin, Jun-Jie Wu, Yong-Qing Peng, Shu-Hang Hu, Hai-Shan Yan, Fan Xiao, Xuan Zhou, Jing Gao, Rui-Hong Wang, Ling Xu, Zhe-Chen Qi

**Affiliations:** a Zhejiang Province Key Laboratory of Plant Secondary Metabolism and Regulation, College of Life Sciences and Medicine, Zhejiang Sci-Tech University, Hangzhou, Zhejiang, China;; b Shanghai Key Laboratory of Plant Functional Genomics and Resources, Shanghai Chenshan Botanical Garden, Shanghai, China

**Keywords:** *Gelsemium elegans*, mitochondrial genome, phylogenetics, toxic plant

## Abstract

*Gelsemium elegans*, endemic to southern Asia, is a highly toxic plant with various medicinal functions. The complete mitochondrial genome of *G. elegans* was sequenced and assembled in this study. The genome is 405,990 bp in length and contains 37 protein-coding genes, 20 tRNA genes, and 3 rRNA genes. The phylogenetic tree based on 15 protein-coding genes common to six mitochondrial genomes in Gentianales support that *G. elegans* of Gelsemiaceae is sister to Apocynaceae.

*Gelsemium elegans* (Gardner & Champ.) Benth., also known as heartbreak grass or ‘Duan Chang Cao’ in Chinese, is a highly poisonous vine in Gelsemiaceae. It is indigenous to south and southeast Asia. *Gelsemium* extract contains toxic alkaloids such as gelsemine, gelsenicine, gelsevirine, and koumine (Zhang et al. 2015). Intoxication of *G. elegans* could cause paralysis, asphyxia, convulsions, and even death due to multiple organ failure in severe conditions (Zhou et al. 2017). Previous studies demonstrated that *G. elegans* had anti-inflammatory, immunomodulating, analgesic, anxiolytic, anti-tumor, and neuropathic pain-relieving properties (Liu et al. 2019). In this study, we report the first complete mitochondrial genome of *G. elegans* which will provide essential genetic data for further genetic improvement and effective use of this medicinally important species.

The leaves of *G. elegans* were collected from Liuzhou, Guangxi, China (GPS: E109°14'43'', N24°20'40'', Voucher No. ZSTU03201, deposited at Herbarium of Zhejiang University). DNA was extracted from its silica dried leaves using DNA Plantzol Reagent (Invitrogen, Carlsbad, CA). The total genomic sequences were generated using the Illumina HiSeq 2500 platform (Illumina Inc., San Diego, CA). In total, about 9.2 G high-quality clean reads (150 bp PE read length) were generated with adaptors trimmed. Firstly, the chloroplast genome of *G. elegans* was firstly assembled following Liu et al. (2017, 2018). Then, sequence of *G. elegans* plastome and a sequence of ATPase subunit 1 (Accession: KF541337) were set as reference in NOVOPlasty (Dierckxsens et al. 2017) for mitochondrion genome *de novo* assembly. Finally, aligning and annotation were conducted by BLAST, GeSeq (Tillich et al. 2017) and GENEIOUS Prime (Biomatters Ltd., Auckland, New Zealand).

The complete mitochondrial genome of *G. elegans* (Accession: MN388837) has a length of 405,990 bp with a typical circle structure and a GC content of 44.4%. The genome contains 37 protein-coding genes, including ones for NADH dehydrogenase (*nad1*, *2*, *3*, *4, 4L, 5, 6, 7, 9*), cytochrome c oxidase (*cox1*, *2*, *3*), cytochrome c biogenesis (*ccmB*, *C*, *Fn*, *Fc*), apocytochrome b (*cob*), ATP synthase (*atp1*, *4*, *8*, *9*), ribosomal proteins (*rpl2*, *5*, *10* and *rps1*, *3*, *4*, *7*, *10*, *12*, *13*, *19*), succinate dehydrogenase(*sdh3*, *4*), maturase and membrane transporter (*mttB* and *matR*). Additionally, the genome also contains 20 tRNA genes coding for 17 amino acids, 3 rRNA genes (*rrn5*, *18*, *26*).

Fifteen mitochondrial specific protein-coding genes (*atp1*, *atp4*, *atp8*, *atp9*, *ccmB*, *ccmC*, *cob*, *cox2*, *mttB*, *nad3*, *nad4*, *nad7*, *rpl5*, *rps13*, *rps3*) from six species were selected to study the phylogenetic placement of *G. elegans* in Gentianales. The sequence alignment was conducted by MAFFT v7.3 (Katoh and Standley 2013). The software IQtree (Nguyen et al. 2015) was used to draw the phylogenetic tree with 5000 bootstrap replicates and TVM + F + G4 model. The phylogenetic tree exhibited that *G. elegans* of Gelsemiaceae was closely related to Apocynaceae in Gentianales (Figure 1).

## Figures and Tables

**Figure 1.**
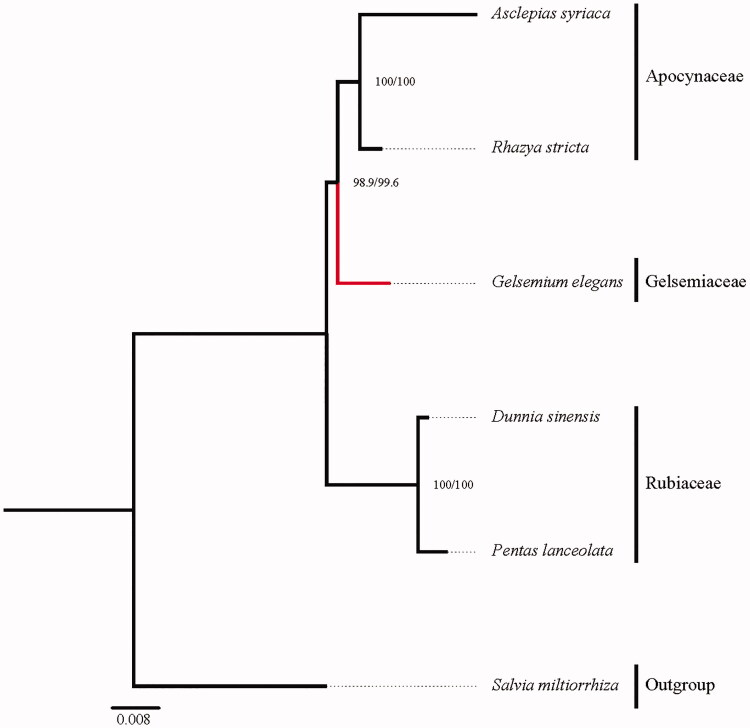
The best maximum-likelihood (ML) phylogram inferred from 6 plant mt genomes based on 15 protein-coding genes. Accession numbers are as follows: *Asclepias syriaca* (KF541337), *Rhazya stricta* (KJ485850), *Salvia miltiorrhiza* (KF177345), *Pentas lanceolate* (KY492150, KY637224, KY637398, KY637630, KY637974, KY638032, KY638090, KY638148, KY638264, KY638495, KY638957, KY639015, KY639073, KY639131, KY639229), and *Dunnia sinensis* (KY492163, KY637237, KY637411, KY637643, KY637987, KY638045, KY638103, KY638161, KY638277, KY638506, KY638970, KY639028, KY639086, KY639144, KY639242).
